# Effectiveness of Multisymptom Support for Better Relief and Alleviation of Common Effects in Perimenopause (EMBRACE PERIMENOPAUSE)

**DOI:** 10.7759/cureus.86091

**Published:** 2025-06-15

**Authors:** Reena Choudhury, Kiran Coelho, Sachin Suryawanshi, Anoop Hajare, Abhijeet Kumar

**Affiliations:** 1 General Medicine, Redkar Hospital and Research Center, Goa, IND; 2 Gynecology, Lilavati Hospital and Research Centre, Mumbai, IND; 3 Medical Services, Emcure Pharmaceuticals Ltd., Mumbai, IND

**Keywords:** ashwagandha (withania somnifera), nutraceuticals, perimenopause, quality of life (qol), vitex agnus castus

## Abstract

Background: Menopause affects 1.5 million women annually, with perimenopause lasting 2-8 years and causing symptoms like hot flashes, sleep disturbances, mood changes, and joint pain. While hormone replacement therapy (HRT) is effective, safety concerns limit its use, leading many women to seek nonhormonal alternatives. Nutraceuticals such as ashwagandha, vitex, and black cohosh offer potential benefits, with 80% of women using supplements. This study evaluates the efficacy and safety of Arth perimenopause multisymptom capsules, providing evidence for nutraceuticals as an alternative to HRT.

Methodology: The Effectiveness of Multisymptom Support for Better Relief and Alleviation of Common Effects in Perimenopause (EMBRACE PERIMENOPAUSE) study was a prospective, observational trial conducted at Redkar Hospital and Research Center, Goa. Thirty perimenopausal women aged 40-48 years with irregular menstrual cycles and at least two menopausal symptoms received one Arth capsule daily for 60 days. Using standardized case report forms, data were collected at screening, baseline, and days 7, 15, 30, and 60. Primary outcomes included changes in menopausal symptoms (Menopause Rating Scale (MRS)), reduction in daily hot flashes, and improvement in breast tenderness. Secondary outcomes assessed the quality of life (QoL), changes in hair and skin texture, patient and physician global assessments, and intervention tolerability. Statistical analyses included Chi-square tests and analysis of variance (ANOVA) (p < 0.05).

Results: At baseline, 100% of patients reported hot flashes, followed by irritability (90%) and night sweating (83%). The mean MRS score decreased from 39 at baseline to 2.74 by day 60 (-92.97%, p < 0.01). By day 60, 93% of patients achieved complete resolution of hot flashes, 88% for sweating, 91% for anxiety, and 90% for depressive mood (p < 0.05). Overall, treatment significantly improved menopausal symptoms, QoL, and patient satisfaction.

Conclusion: Arth Perimenopause Multisymptom Support capsules demonstrated significant efficacy in alleviating perimenopausal symptoms while improving QoL. This nonhormonal, well-tolerated nutraceutical represents a promising alternative for managing perimenopause-related discomfort.

## Introduction

Each year, 1.5 million women undergo the menopause transition, often accompanied by challenging symptoms like hot flashes, vaginal dryness, reduced libido, insomnia, fatigue, and joint pain [1]. Perimenopause is when initial clinical signs of physiological changes emerge, marking the start of the transition to menopause. This stage generally begins two to eight years before menopause, often occurring around the mid-forties [2]. Recent epidemiological data show that 30%-70% of premenopausal women experience hot flashes, though they are generally mild at this stage of life [3]. The prevalence rises to around 39% as women enter the early menopause transition and nearly doubles, reaching a cumulative rate of 67%, as reported in the Study of Women’s Health Across the Nation (SWAN) study [4].

Hormone replacement therapy (HRT) is regarded as the most effective option for achieving relief from various menopausal symptoms [5]. Although HRT is widely recognized as the gold standard treatment for women with menopausal symptoms, reports indicate that fewer than 30% of menopausal women use HRT, and only 15% remain on the therapy long-term [6,7]. In addition, many women decline HRT due to various concerns, including the fear of cancer and potential side effects like weight gain [8]. In recent years, nutraceuticals have become highly popular compared to HRT, largely due to their purported effectiveness in relieving menopausal symptoms [9]. Herbal remedies such as isoflavones, black cohosh, red clover, pollen extracts, and others may be utilized for symptomatic menopausal women. Surveys in the US and Britain reveal that 80% of peri- and postmenopausal women have used dietary supplements, either currently or in the past [10].

Ashwagandha is a beneficial option for managing symptoms during perimenopause, offering a range of effects, such as anti-stress, anti-aging, immune support, anti-inflammatory, cognition enhancement, anxiety relief, adaptogenic properties, and sleep promotion [11]. Similarly, Vitex has been widely used in traditional medicine, with preliminary studies indicating its effectiveness in alleviating menopausal symptoms [12]. Black cohosh has also shown positive effects in reducing hot flashes and improving other symptoms, such as sleep quality [13]. Furthermore, a randomized trial found black cohosh to be as effective as tibolone for treating menopausal symptoms, with the herbal group demonstrating a superior safety profile [14]. Nonhormonal therapies are continually evolving, and many women seek a “natural” approach to manage menopausal symptoms. Nutraceuticals, medicinally active compounds derived from food or plants, offer a promising alternative to this approach [15].

This study addresses the need for safer, nonhormonal options to manage perimenopausal symptoms, as many women seek alternatives to HRT due to associated risks. Nutraceuticals offer a promising solution with a favorable safety profile and potential to alleviate a range of perimenopausal symptoms, including mood changes, sleep disturbances, and vasomotor symptoms. This investigation aims to assess the efficacy of Arth Perimenopause Multisymptom capsules for nutraceutical support, providing evidence to guide safe and effective symptom relief during perimenopause.

## Materials and methods

Study design

The Effectiveness of Multisymptom Support for Better Relief and Alleviation of Common Effects in Perimenopause (EMBRACE PERIMENOPAUSE) was a prospective, observational, real-world evidence study conducted at Redkar Hospital and Research Center, Dargalim, Goa. The study documents were reviewed, and the protocol was approved by Redkar Hospital and Research Center Institutional Ethics Committee (Reg. No. ECR/902/Inst/GA/2018/RR-21), Goa. The study was conducted as per the Protocol, the Declaration of Helsinki, Good Clinical Practice (GCP) guidelines, and the Indian Council of Medical Research (ICMR) guidelines for medical research in humans. Before the screening, the principal investigator explained the study to the participants. Informed consent was obtained from those willing to participate. The study was initiated on July 26, 2024, after receiving EC approval, and was concluded on November 9, 2024.

Participants

The inclusion criteria for this study were perimenopausal women aged 40-48 years who had irregular menstrual cycles and reported experiencing at least two menopausal symptoms, such as sleep disturbances, mood changes, hot flashes, night sweats, fatigue, reduced energy, changes in sexual or urinary function, decreased libido, hair fall, dry skin, or vaginal changes. Eligible participants also exhibited serum follicle-stimulating hormone (FSH) levels above 20 IU/L and serum luteinizing hormone (LH) levels within the 5-25 IU/L range. Additionally, participants agreed to comply with all aspects of the study protocol.

The exclusion criteria for this study were participants who had taken herbal supplements or multivitamins within the past month, were currently using or planning to start conventional hormone replacement therapies during the study, or had absent menstrual periods. Participants were excluded if they had allergies to any product ingredients or a history of uterine fibroids, endometriosis, polycystic ovarian syndrome (PCOS), or abnormal pap smear results. Additionally, participants with a history of breast, endometrial, or cervical cancer; undiagnosed abnormal vaginal bleeding; thromboembolic disease; liver or kidney disease; or other significant illnesses were ineligible. Exclusions also applied to those with uncontrollable hypertension, diabetes, thyroid diseases, diabetic neuropathy, malignant cancers, mental health conditions (including depression), or those planning invasive medical procedures during the study period. Lastly, women who were currently taking or had taken oral contraceptives within the past three months were also excluded.

Study product

Ishaanav Nutraceuticals Pvt. Ltd. manufactures the Arth Perimenopause Multisymptom Support capsules in Selaqui, Dehradun, Uttarakhand, and is marketed by Emcure Pharmaceuticals Ltd. Subjects were instructed to take Arth Perimenopause Multisymptom Support capsules (ashwagandha KSM-66: 300 mg, chasteberry extract (*Vitex agnus-castus*): 50 mg; isoflavones 40% (from soybean extract): 50 mg; black cohosh root extract: 30 mg; magnesium (as magnesium glycinate): 31.25 mg; vitamin B6: 1.9 mg; vitamin B1: 1.4 mg; vitamin D (ergocalciferol): 15 mcg; vitamin B12 (cyanocobalamin): 2.2 mcg) once daily for two months. Patients were advised not to take other ayurvedic/herbal/homoeopathic dietary supplements or alternative therapies during treatment. A record of these medications was maintained.

Outcomes

**Figure 1:**
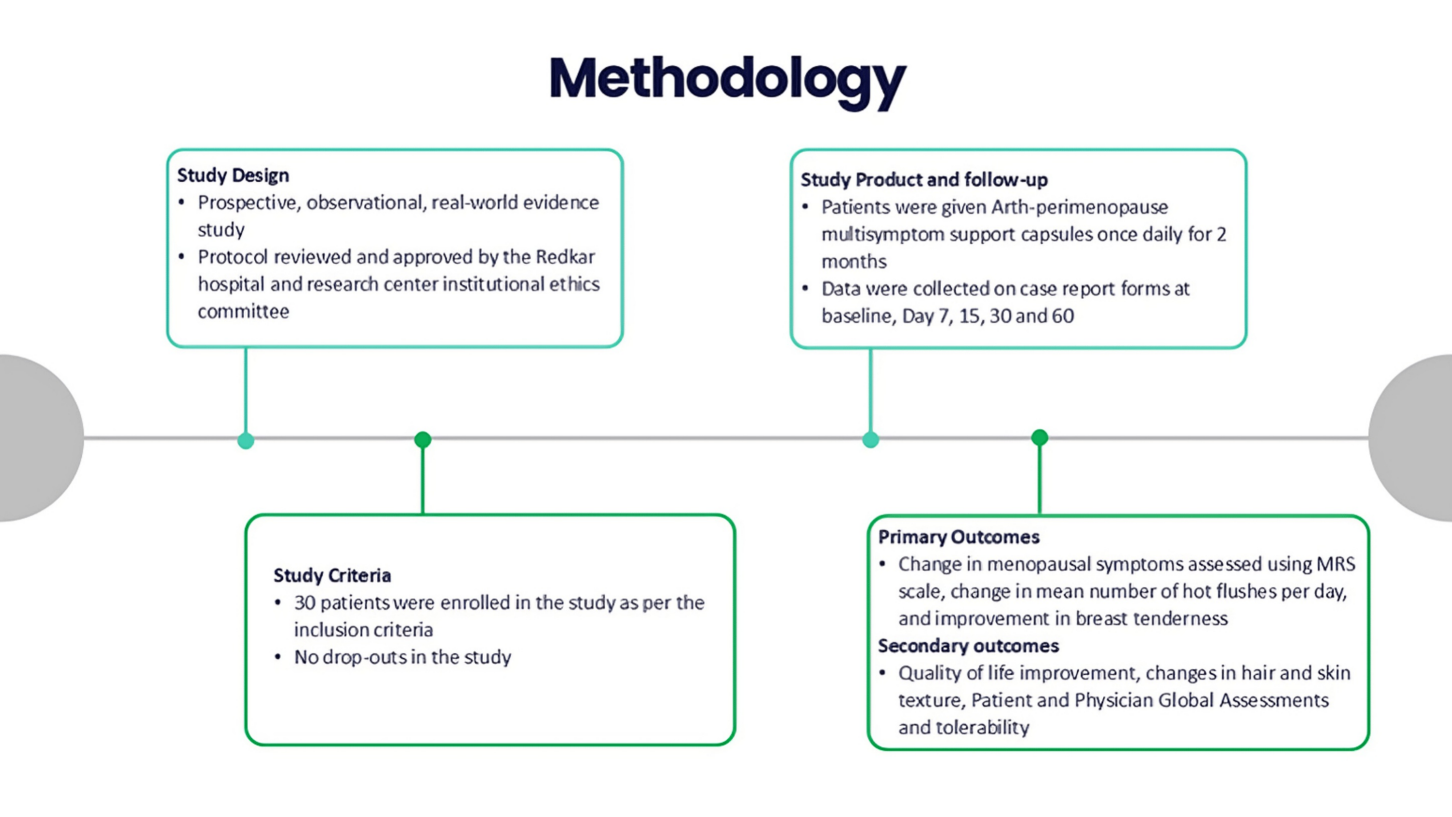
Overview of the study methodology and outcome measures MRS: Menopause Rating Scale

Data were collected using standardized case report forms at screening: baseline, day 7, 15, 30, and 60. The primary outcomes included changes in menopausal symptoms, assessed using the Menopause Rating Scale (MRS), a five-point Likert scale ranging from 0 (none) to 4 (very severe). The MRS comprises 11 symptoms or complaints, each rated from 0 to 4 based on severity, as perceived by the women completing the scale. It consists of three domains: psychological, somato-vegetative, and urogenital. The total score across these domains is categorized as follows: 0-4 (no or little symptoms), 5-8 (mild), 9-16 (moderate), and 17 or higher (severe) [16]. Additional outcomes included a reduction in the mean number of daily hot flashes and an improvement in breast tenderness, both assessed using the same five-point Likert scale. Secondary outcomes included quality of life (QoL) improvement using a five-point Likert scale (0 = not at all bothered to 4 = extremely bothered) and changes in hair and skin texture using a seven-point scale ranging from -3 (greatly worsened) to +3 (significantly improved). Additional assessments included the Patient and Physician Global Assessments and the overall tolerability of the intervention. These outcomes were collectively designed to evaluate symptom relief, patient satisfaction, and tolerability comprehensively. Patient satisfaction was assessed based on overall improvement after the treatment, using a scale from 1 (not satisfactory) to 4 (very satisfactory). Similarly, physicians rated the overall improvement in the patient’s condition on the same 1 to 4 scale since the start of treatment (Figure 1) [17].

Sample size and statistical analysis

The study included a sample size of 30 participants. Descriptive statistics were employed to summarize patient demographics, baseline characteristics, and outcomes. For categorical variables, the Chi-square test was utilized to assess associations, while analysis of variance (ANOVA) was applied to evaluate continuous variables. A p-value of less than 0.05 was considered statistically significant for all analyses.

## Results

A total of 30 patients were enrolled, with a mean age of 42.23 ± 2.18 years. All the patients completed the study. The mean follicle-stimulating hormone (FSH) and luteinizing hormone (LH) were 28.7 IU/L and 21.5 IU/L, respectively. The most common comorbidity was the presence of hypothyroidism (7/30) (Table 1).

**Table 1:** Baseline characteristics of the study participants FSH: follicle-stimulating hormone; LH: luteinizing hormone; IU/L: international units per liter; SD: standard deviation

Parameter	Baseline characteristics (n = 30)
Age: mean + SD	42.23 ± 2.18 years
Mean FSH	28.7 IU/L
Mean LH	21.5 IU/L
Comorbidity n(%) hypothyroidism	7 (23%)

**Figure 2:**
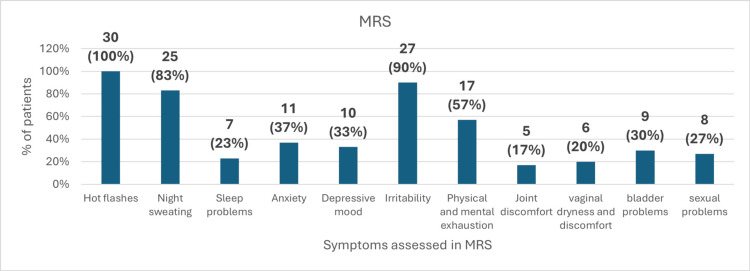
Common symptoms experienced by the patients MRS: Menopause Rating Scale

At baseline, the most commonly observed symptom was hot flashes, reported by all patients (100%). This was followed by irritability in 90% of patients and night sweating in 83%. Other symptoms included physical and mental exhaustion (57%), anxiety (37%), depressive mood (33%), bladder problems (30%), sexual problems (27%), sleep problems (23%), vaginal dryness and discomfort (20%), and joint discomfort (17%) (Figure 2).

Change in MRS

**Figure 3:**
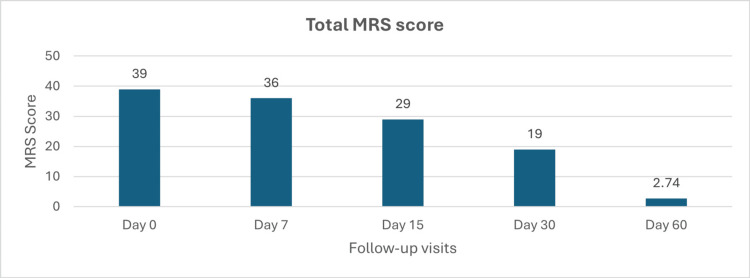
Change in the total MRS score MRS: Menopause Rating Scale

The mean MRS score at baseline was 39, indicating moderate to severe menopausal symptoms. A steady decline in MRS scores was observed throughout the study, with scores decreasing to 36 by day 7, 29 by day 15, and 19 by day 30. By day 60, the mean MRS score had further reduced to 2.74, suggesting a substantial reduction in menopausal symptom severity (Figure 3).

Percentage change in the total MRS from baseline

The percentage change in total MRS scores from baseline to various time points is given in Table 2. From baseline to day 7, there was a reduction of -7.69%, followed by a change of -25.64% by day 15. The change continued to increase by -51.28% at day 30. Notably, by day 60, the reduction reached -92.97%, with a statistically significant p-value of <0.01.

**Table 2:** Change in the total MRS from baseline to the different time points MRS: Menopause Rating Scale The p-values were determined using an individual paired t-test

Total score	Change from baseline to day 7	Change from baseline to day 15	Change from baseline to day 30	Change from baseline to day 60
% change	-7.69%	-25.64%	-51.28%	-92.97%
p-value	p > 0.05	p > 0.05	p = 0.039	p < 0.01
t-value	0.897	1.072	2.781	4.762

Percentage change in total MRS between two consecutive visits

The percentage change in total MRS between two consecutive visits is shown in detail in Table 3.

**Table 3:** Percentage change in total MRS between two consecutive visits​​​​​​​ MRS: Menopause Rating Scale The p-values were determined using an individual paired t-test

	Change from baseline to day 7	Change from day 7 to day 15	Change from day 15 to day 30	Change from day 30 to day 60
% Change between two visits	-7.69%	-19.44%	-34.48%	-85.57%
p-value	p > 0.05	p > 0.05	p > 0.05	p = 0.031
t-value	0.897	1.126	1.342	2.981

Symptom-wise MRS score at each visit

**Figure 4:**
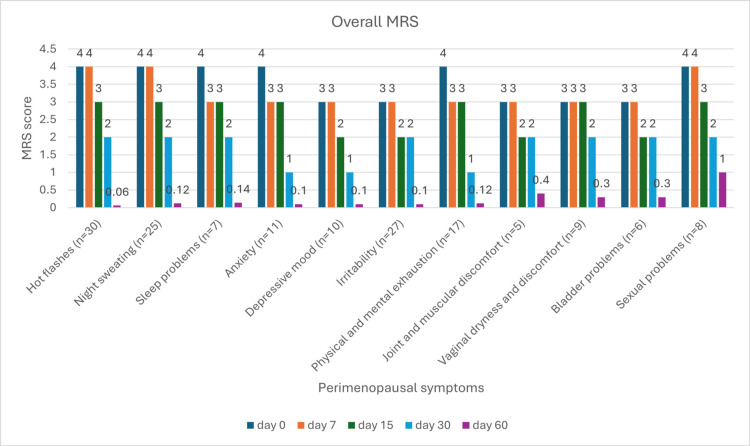
Change in symptom-wise MRS score MRS: Menopause Rating Scale

At baseline (day 0), several symptoms, including hot flashes, night sweating, and physical/mental exhaustion, were characterized by high severity, each scoring a mean of 4 on the MRS scale. Throughout the intervention period, a progressive reduction in symptom intensity was observed across most symptom categories. By day 60, significant improvements were noted, particularly in hot flashes, night sweating, and physical/mental exhaustion, which showed substantial decreases. Moreover, symptoms such as vaginal dryness and discomfort, bladder issues, and sexual dysfunction, which initially exhibited lower prevalence and severity, also experienced improvement by the end of the study (Figure 4).

Percentage reduction in symptom-wise in the MRS score from baseline

The data presented in Table 4 highlights the progression of various symptoms over time, from baseline to day 7, day 15, day 30, and day 60. Symptoms such as hot flashes, night sweating, anxiety, and depressive mood show significant improvements by day 30, with 50%-75% of participants reporting improvement. A notable reduction in joint discomfort, physical and mental exhaustion, and irritability is observed by day 60, with most participants achieving 87%-97% improvement. Symptoms like sexual problems, vaginal dryness, and bladder problems demonstrate consistent improvements, reaching 75%-90% by day 60.

**Table 4:** Percentage change from baseline in symptom severity over time ANOVA: analysis of variance; *p < 0.05 Repeated measures ANOVA test was used to test significance at multiple follow-ups. p < 0.05 was considered a significant change in the values

	Change from baseline to day 7	Change from baseline to day 15	Change from baseline to day 30	Change from baseline to day 60	F-value	p-value
Hot flashes (n = 30)	0%	25%	50% ^*^	99% ^*^	136.18	<0.05
Night sweating (n = 26)	0%	25%	50%	97% ^*^	118.29	<0.05
Sleep problems (n = 7)	25%	25%	50%	97% ^*^	78.88	<0.05
Anxiety (n = 11)	25%	25%	75% ^*^	98%	82.74	<0.05
Depressive mood (n = 10)	0%	33%	67%	97% ^*^	92.18	<0.05
Irritability (n = 27)	0%	33%	33%	97% ^*^	110.92	<0.05
Physical and mental exhaustion (n = 17)	25% ^*^	25%	75% ^*^	97% ^*^	102.78	<0.05
Joint and muscular discomfort (n = 5)	0%	33%	33%	87% ^*^	65.82	<0.05
Vaginal dryness and discomfort (n = 9)	0%	0%	33%	90% ^*^	74.28	<0.05
Bladder problems (n = 6)	0%	33%	33%	90% ^*^	69.28	<0.05
Sexual problems (n = 8)	0%	25%	50%	75% ^*^	70.27	<0.05

The treatment of perimenopausal symptoms with Arth Perimenopause Multisymptom Support capsules showed significant improvement in symptom severity over 60 days.

Percentage reduction in symptom-wise MRS score between two consecutive visits​

The percentage reduction in symptom-wise MRS score between two consecutive visits is detailed in Table 5.

**Table 5:** Percentage reduction in symptom-wise MRS score between two consecutive visits MRS: Menopause Rating Scale; ANOVA: analysis of variance; *p < 0.05 Repeated-measures ANOVA test was used to test significance at multiple follow-ups. p < 0.05 was considered a significant change in the values

	Change from baseline to day 7	Change from day 7 to day 15	Change from day 15 to day 30	Change from day 30 to day 60	F-value	p-value
Hot flashes (n = 30)	0%	25%^*^	33% ^*^	97% ^*^	142.87	<0.05
Night sweating (n = 26)	0%	25%	33%	94%^*^	138.92	<0.05
Sleep problems (n = 7)	25%	0%	33%	93%^*^	78.92	<0.05
Anxiety (n = 11)	25%	0%	67%	90% ^*^	85.82	<0.05
Depressive mood (n = 10)	0%	33%	50%	90% ^*^	92.81	<0.05
Irritability (n = 27)	0%	33%	0%	95% ^*^	108.88	<0.05
Physical and mental exhaustion (n = 17)	25% ^*^	0%	67% ^*^	88% ^*^	100.26	<0.05
Joint and muscular discomfort (n = 5)	0%	33%	0%	80% ^*^	61.98	<0.05
Vaginal dryness and discomfort (n = 9)	0%	0%	33%	85% ^*^	73.71	<0.05
Bladder problems (n = 6)	0%	33%	0%	85% ^*^	79.18	<0.05
Sexual problems (n = 8)	0%	25%	33%	50%	71.90	<0.05

Hot Flashes (n = 30)

**Figure 5:**
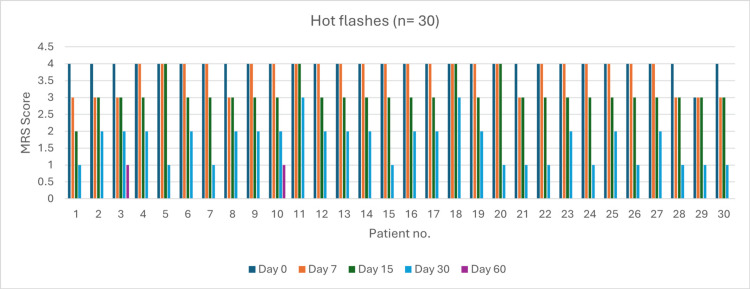
Change in hot flash severity (MRS) over time in perimenopausal patients MRS: Menopause Rating Scale

At baseline (day 0), 29 patients experienced very severe symptoms. By day 60, 28 patients (93%) achieved complete resolution. Severity reduction was 24.13% by day 7 (p > 0.05), 86.20% by day 15 (p = 0.04), and 100% by day 30 (p = 0.038). Overall symptom resolution at day 60 was 93% (p < 0.05) (Figure 5).

Sweating (n = 25)

**Figure 6:**
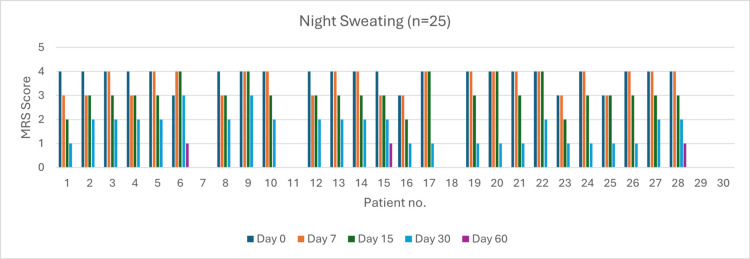
Change in night sweating severity (MRS) over time in perimenopausal patients MRS: Menopause Rating Scale

Initially, 21 patients reported very severe symptoms. By day 60, 22 patients (88%) experienced complete resolution. The severity reduction was 23.80% by day 7 (p > 0.05), 76.20% by day 15 (p > 0.05), and 100% by day 30 (p = 0.047). The overall resolution at day 60 was 88% (p < 0.05) (Figure 6).

Sleep Problems (n = 7)

**Figure 7:**
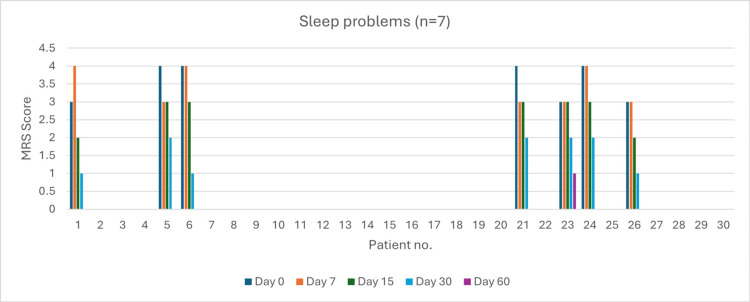
Change in sleep problem severity (MRS) over time in perimenopausal patients MRS: Menopause Rating Scale

Four patients reported very severe sleep problems at baseline. By day 60, all symptoms were entirely resolved in six patients out of seven (86%). The severity reduction was 25% by day 7 (p = 0.03) and 100% by day 15 (p = 0.03). At day 60, 86% resolution was statistically significant (p < 0.05) (Figure 7).

Anxiety (n = 11)

**Figure 8:**
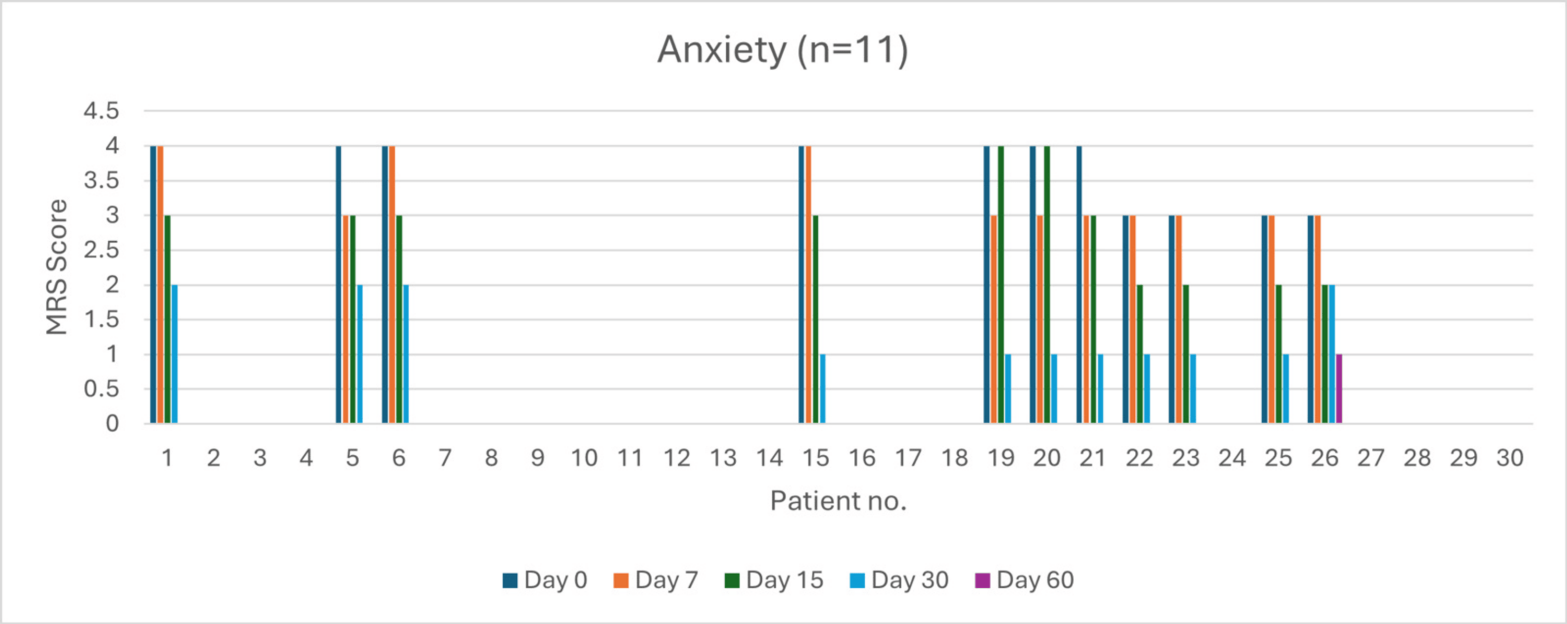
Change in anxiety severity (MRS) over time in perimenopausal patients MRS: Menopause Rating Scale

At baseline, seven patients experienced very severe anxiety. By day 60, 90% of patients had achieved complete resolution. The severity reduction was 57.14% by day 7 (p > 0.05), 71.42% by day 15 (p > 0.05), and 100% by day 30 (p = 0.0485). The overall symptom resolution rate was 91% (p < 0.05) (Figure 8).

Depressive Mood (n = 10)

**Figure 9:**
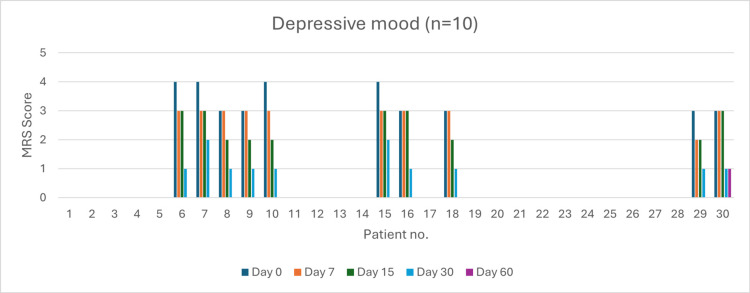
Change in depressive mood severity (MRS) over time in perimenopausal patients MRS: Menopause Rating Scale

At baseline, four patients experienced very severe depressive symptoms. By day 60, complete resolution was observed in 90% of patients (p < 0.05). Severity reduction was 100% by day 7 (p > 0.05), day 15 (p > 0.05), and day 30 (p > 0.05) (Figure 9).

Irritability (n = 27)

**Figure 10:**
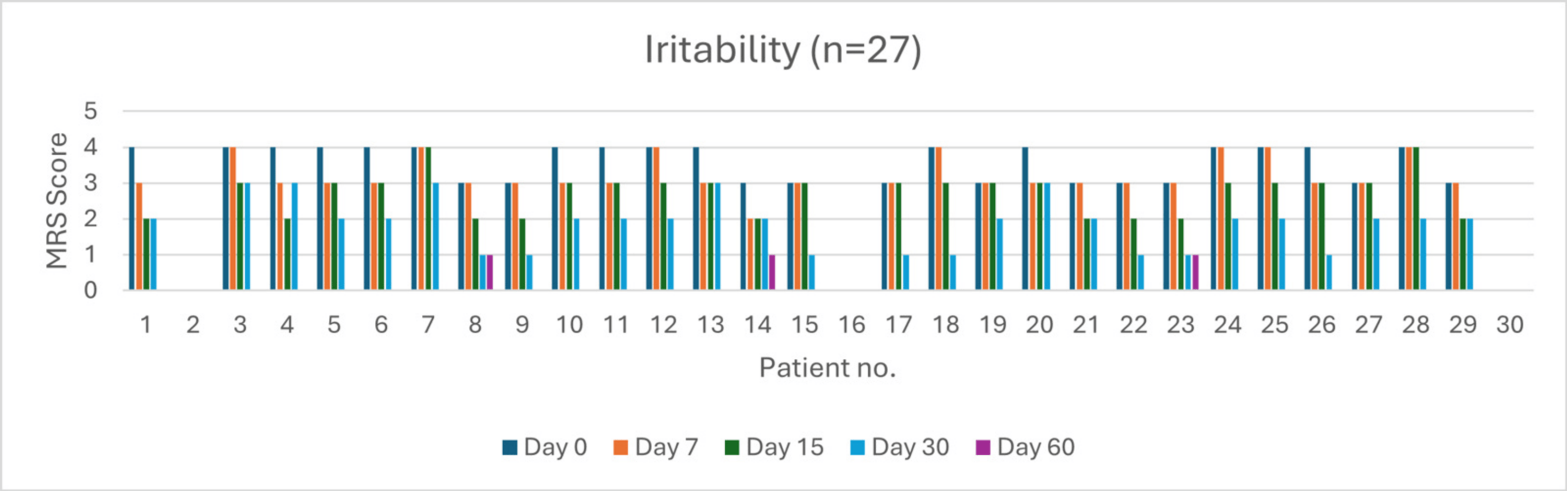
Change in irritability severity (MRS) over time in perimenopausal patients MRS: Menopause Rating Scale

Initially, 16 patients reported very severe irritability. By day 60, 24 patients (89%) experienced complete resolution. Severity reduction was 56.25% by day 7 (p > 0.05), 87.50% by day 15 (p > 0.05), and 100% by day 30 (p = 0.048). Overall resolution at day 60 was significant (p < 0.05) (Figure 10).

Physical and Mental Exhaustion (n = 17)

**Figure 11:**
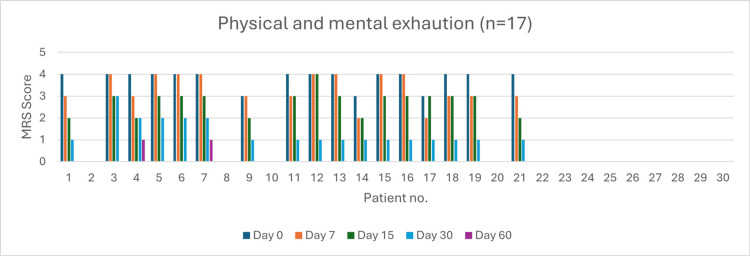
Change in physical and mental exhaustion severity (MRS) over time in perimenopausal patients MRS: Menopause Rating Scale

At day 0, 14 patients experienced very severe exhaustion. By day 60, complete symptom resolution was observed in 15 patients (88%). Severity reduction was 42.85% by day 7 (p = 0.02), 92.85% by day 15 (p = 0.04), and 100% by day 30 (p = 0.037) (Figure 11).

Joint and Muscular Discomfort (n = 5)

**Figure 12:**
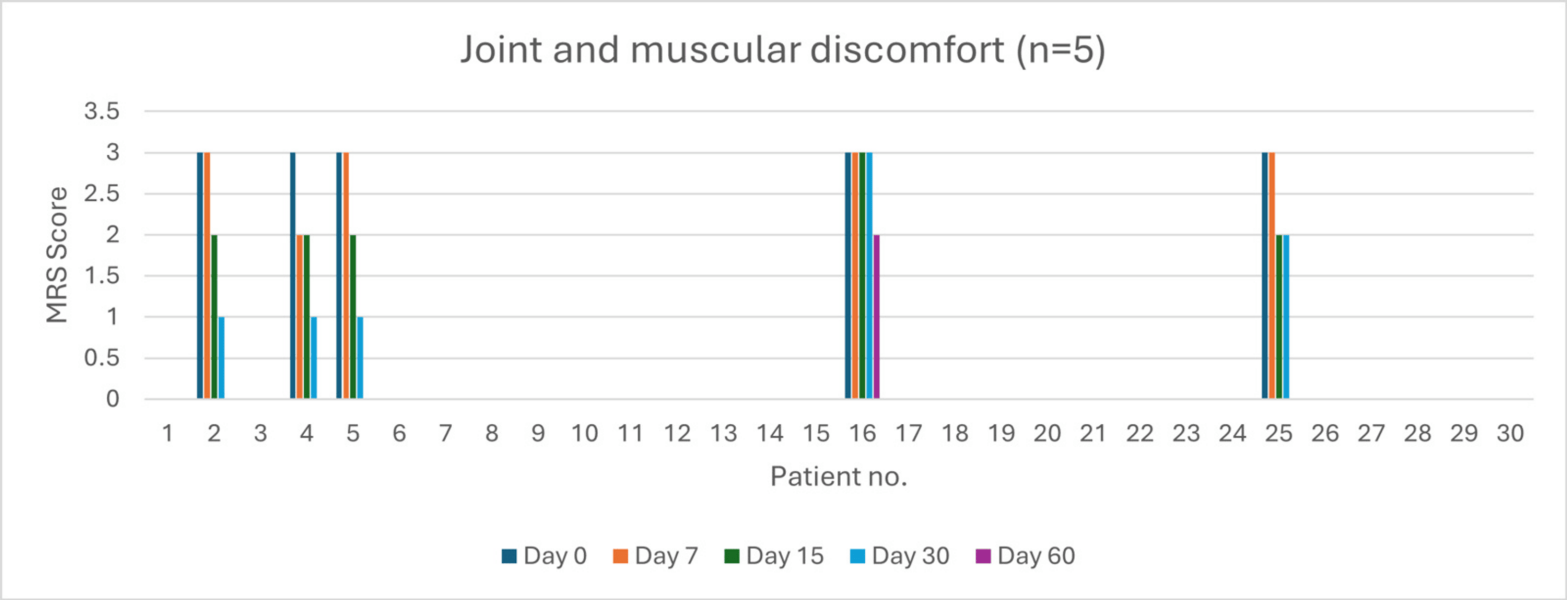
Change in joint and muscular discomfort severity (MRS) over time in perimenopausal patients MRS: Menopause Rating Scale

At baseline, five patients experienced severe discomfort. By day 60, four patients achieved complete resolution (80%, p < 0.05). Severity reduction was 20% by day 7 (p > 0.05), 80% by day 15 (p > 0.05), and 90% by day 30 (p > 0.05) (Figure 12).

Vaginal Dryness (n = 6)

**Figure 13:**
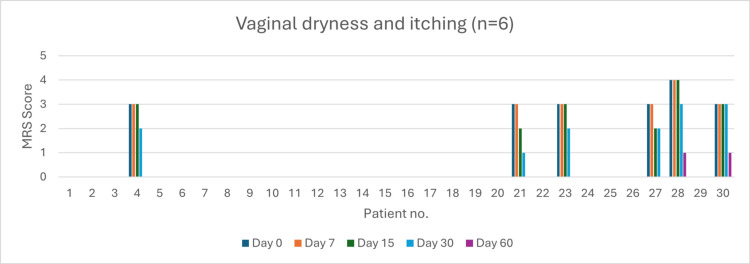
Change in vaginal dryness and itching severity (MRS) over time in perimenopausal patients MRS: Menopause Rating Scale

At baseline, one patient had very severe vaginal dryness, and five had severe symptoms. By day 60, 66.66% of patients achieved complete resolution (p < 0.05). No significant reduction in severity was observed by day 7 (0%, p > 0.05) and day 15 (0%, p > 0.05). By day 30, a 100% severity reduction was noted (p > 0.05) (Figure 13).

Bladder Problems (n = 9)

**Figure 14:**
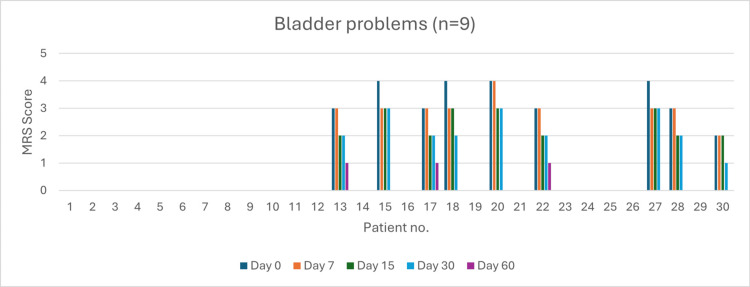
Change in bladder problem severity (MRS) over time in perimenopausal patients MRS: Menopause Rating Scale

Four patients reported very severe bladder problems at baseline. By day 60, six patients achieved complete resolution (66%). Severity reduction was 75% by day 7 (p > 0.05) and 100% by day 15 (p > 0.05) and day 30 (p > 0.05) (Figure 14).

Sexual problems (n = 8)

**Figure 15:**
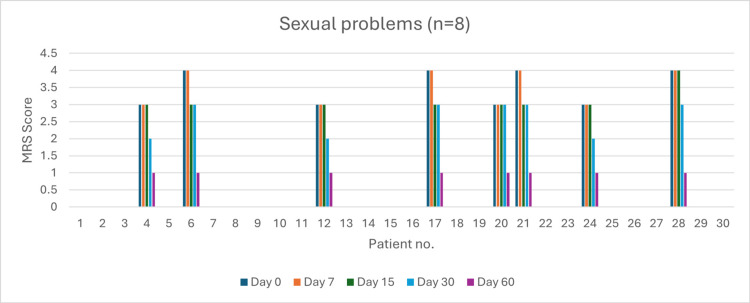
Change in sexual problem severity (MRS) over time in perimenopausal patients MRS: Menopause Rating Scale

At day 0, four patients reported very severe sexual problems, and four reported severe symptoms. By day 60, symptoms were mild in all the patients (p < 0.05). Severity reduction was 0% by day 7 (p > 0.05), 75% by day 15 (p > 0.05), and 100% by day 30 (p > 0.05) (Figure 15).

Hot flashes

Change in the Mean Number of Hot Flashes Per Day

**Figure 16:**
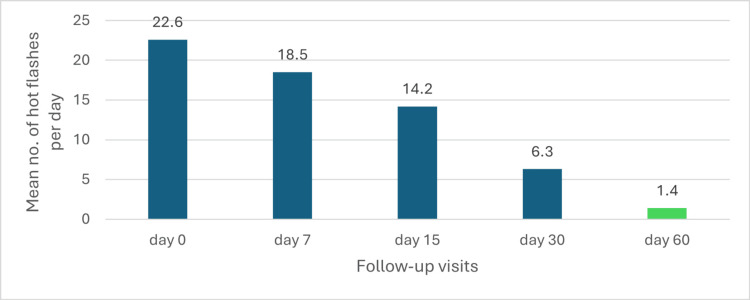
Overall mean number of hot flashes per day

The overall change in the mean number of hot flashes per day is shown in Figure 16.

**Figure 17:**
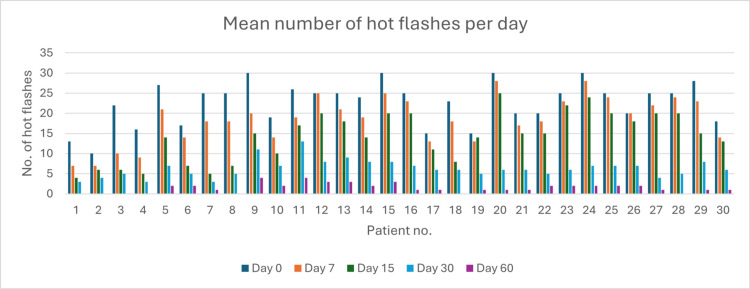
Change in hot flashes episodes over time in perimenopausal patients

Treatment significantly reduced the mean number of hot flashes per day, decreasing from 22.6 at baseline to 18.5 on day 7 and 14.2 on day 15. By day 30, a 72.1% reduction was observed, with the mean dropping to 6.3 hot flashes per day (p < 0.05). This improvement continued through day 60, with a 93.8% reduction from baseline, reaching 1.4 hot flashes per day (p < 0.05). These findings demonstrate the treatment's effectiveness, with statistically significant improvements achieved by day 30 and sustained through day 60, as depicted in the accompanying graph (Figure 17).

Improvement in breast tenderness

At baseline, 22 patients (73%) reported experiencing breast tenderness. After 60 days of treatment, all patients demonstrated significant improvement in breast tenderness (p < 0.05).

Improvement in QoL

**Figure 18:**
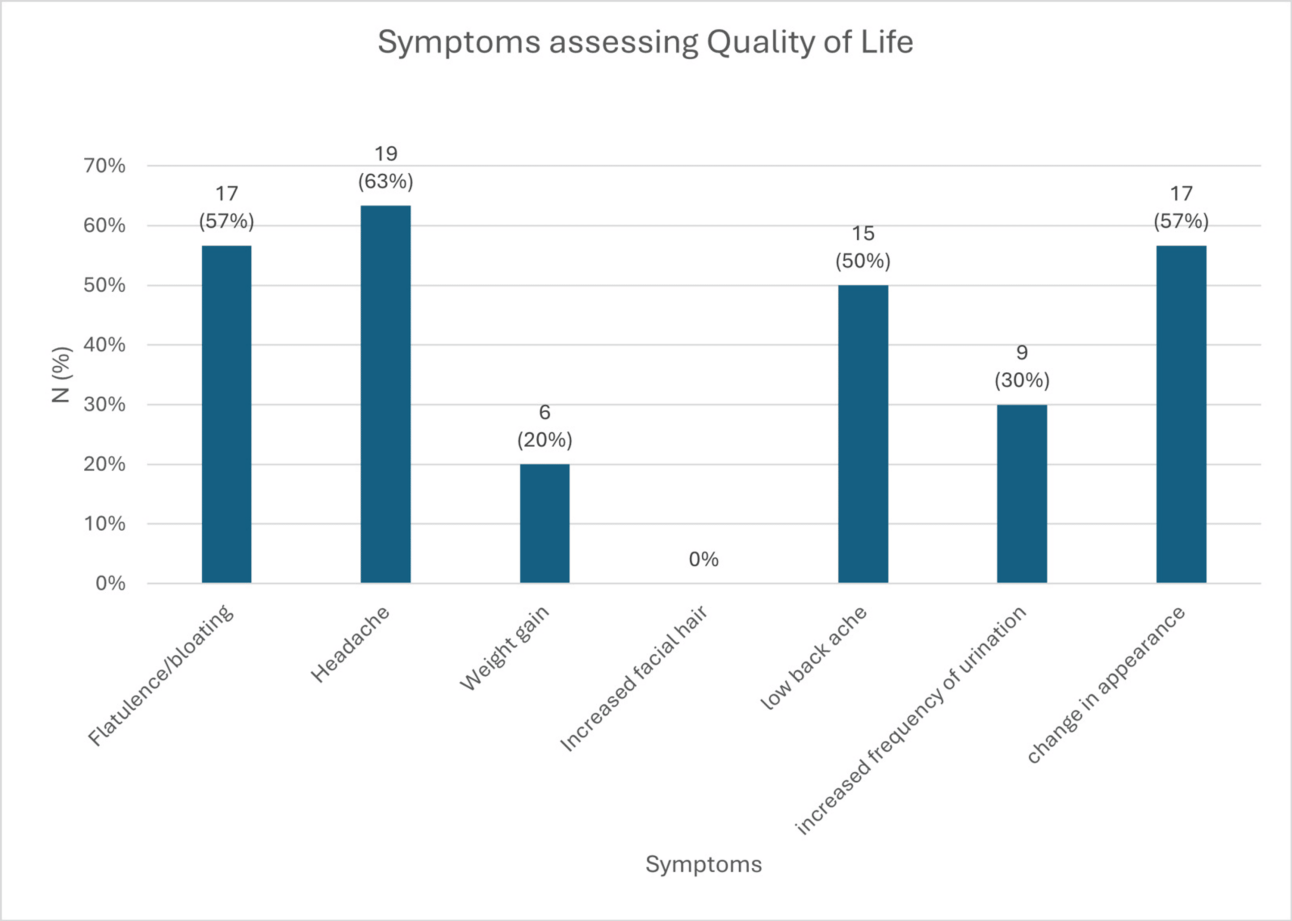
Symptoms assessing for the quality of life

At baseline, patients reported various symptoms, including headache (63%), flatulence/bloating (57%), changes in appearance (57%), low backache (50%), increased frequency of urination (30%), weight gain (20%), and no reports of increased facial hair. Significant improvement in QoL was observed after 30 days of treatment (p < 0.05), with continued improvement through day 60 (p < 0.05, baseline to day 60) (Figure 18).

Improvement in hair and skin texture

None of the patients reported changes in hair characteristics, including texture, shine, dryness, coverage, brittleness, or overall hair appearance. Similarly, no changes were reported in skin characteristics, including texture, tone, fine lines, hydration, or overall skin appearance.

Patient Global Assessment

At the end of the treatment, the mean Patient Global Assessment score was 3.5. A majority of patients (76.66%) rated their treatment experience as "very satisfactory" (score of 4), reflecting high levels of patient satisfaction. The scoring scale ranged from 1 ("not satisfactory") to 4 ("very satisfactory").

Physician Global Assessment

At the end of the treatment, the mean Physician Global Assessment score was 4, with all physicians (100%) rating the treatment outcome as "very satisfactory" (score of 4). The scoring scale ranged from 1 ("not satisfactory") to 4 ("very satisfactory").

## Discussion

Perimenopause affects a woman’s QoL due to physical and psychological symptoms. Nutraceuticals, including herbal remedies like ashwagandha, vitex, and black cohosh, have gained popularity as effective nonhormonal options for managing perimenopausal symptoms. These natural compounds offer a promising solution with a favorable safety profile, addressing symptoms such as hot flashes, mood changes, and sleep disturbances. This study highlights the potential of Arth Perimenopause Multisymptom Support capsules as a safe and effective alternative for alleviating perimenopausal symptoms.

The findings of this study demonstrate the significant efficacy of Arth Perimenopause Multisymptom Support capsules in alleviating a wide range of perimenopausal symptoms. The treatment resulted in marked and statistically significant improvements across several domains, including vasomotor symptoms (hot flashes, sweating), psychological symptoms (anxiety, depressive mood, irritability), and physical symptoms (sleep problems, joint discomfort, and exhaustion). By day 60, all patients experienced complete resolution of hot flashes, sweating, and sleep problems, with notable improvements observed as early as day 7. The rapid resolution of vasomotor symptoms, particularly hot flashes, highlights the potent efficacy of the treatment in addressing one of the most common and distressing symptoms reported by perimenopausal women. By day 60, a 100% reduction in symptom severity was achieved for hot flashes, with significant improvements observed as early as day 15 (86.2% reduction; p < 0.05). These results are consistent with existing evidence suggesting that targeted therapies can effectively manage thermoregulatory instability associated with menopause.

In addition to vasomotor symptoms, the treatment significantly improved psychological well-being. Anxiety, depressive mood, and irritability showed substantial reductions, with most patients achieving complete resolution by day 30 or day 60. This suggests that the formulation may address the underlying neuroendocrine changes associated with perimenopause, including fluctuations in serotonin and estrogen levels, which are implicated in mood disturbances.

Physical symptoms, including joint discomfort and exhaustion, also showed significant improvement, with complete resolution in all patients by day 60. The improvement in QoL, as evidenced by patient-reported outcomes, further highlights the holistic benefits of the treatment. Similarly, an eight-week study by Gopal et al. found that ashwagandha supplementation significantly reduced total MRS scores (p < 0.0001) compared to placebo. Ashwagandha also considerably lowered total menopause-specific quality of life (MENQoL) scores (p < 0.0001), increased serum estradiol (p < 0.0001), and reduced serum FSH (p < 0.0001) and LH (p < 0.05). This suggests that ashwagandha root extract may be a safe, effective option for relieving mild to moderate perimenopausal symptoms in women [18]. In another eight-week study by Naseri et al., results indicated a significant reduction in total scores for menopause symptoms, anxiety, and vasomotor dysfunction in the vitex group following the intervention [19]. Similarly, in the 12-week study by Rattanatantikul et al., nutraceutical supplementation significantly reduced hot flashes and sweating (p < 0.0001), sleep problems (p < 0.0005), depressed mood (p = 0.0004), and irritability (p < 0.0003) compared to the placebo group. A nutraceutical containing a blend of four medicinal herbs, soy isoflavone, black cohosh, chasteberry, and evening primrose oil extracts, was effective and safe in improving menopausal symptoms [20].

A mean Patient Global Assessment score of 3.5 and a 76.66% "very satisfactory" rating reflect high levels of patient satisfaction. Similarly, a perfect Physician Global Assessment score of 4 (100%) emphasizes the clinical efficacy of the intervention. Interestingly, the treatment did not induce changes in hair or skin texture, characteristics, or appearance, indicating its favorable safety profile. The absence of adverse effects on these aspects is particularly reassuring for perimenopausal women, who often experience concerns about aging-related changes. These findings collectively indicate that Arth Perimenopause Multisymptom Support capsules offer a well-tolerated, effective, and comprehensive solution for managing the multifaceted symptoms of perimenopause, significantly improving patients' QoL and overall satisfaction.

While the study demonstrates promising results, certain limitations highlight valuable opportunities for future research. The absence of a placebo or control group limits causal inference, underscoring the need for future randomized, placebo-controlled trials to strengthen the evidence base. With a sample size of 30 patients, the findings provide an excellent foundation, but larger, multicenter studies will further enhance statistical power and generalizability. Additionally, the two-month study duration, while sufficient to observe significant improvements, leaves scope for long-term evaluations. Future research should focus on extended follow-up periods to assess sustained efficacy and long-term safety, ensuring comprehensive insights into the intervention’s benefits.

## Conclusions

The findings of the EMBRACE PERIMENOPAUSE study demonstrate the efficacy and safety of Arth Perimenopause Multisymptom Support capsules in alleviating perimenopausal symptoms. Significant improvements were observed in vasomotor, psychological, and physical symptoms, including complete resolution of hot flashes, sweating, anxiety, depressive mood, irritability, and sleep disturbances by day 60 in the majority of patients. These results were consistent with statistically significant changes as early as day 15 for several symptoms, highlighting the rapid efficacy of the treatment.

Additionally, the intervention was associated with improved QoL and high levels of patient and physician satisfaction, as evidenced by the Patient and Physician Global Assessment scores. Importantly, the study reported no adverse changes in hair or skin texture, underscoring the safety profile of the formulation.

Overall, this study supports the use of Arth Perimenopause Multisymptom Support capsules as a comprehensive, nonhormonal, and natural therapeutic option for alleviating perimenopausal symptoms, offering significant benefits for women seeking effective symptom relief during this transitional phase of life. Further studies with larger sample sizes and longer durations are recommended to validate these findings and explore additional benefits.
